# The Study of HIV and Antenatal Care Integration in Pregnancy in Kenya: Design, Methods, and Baseline Results of a Cluster-Randomized Controlled Trial

**DOI:** 10.1371/journal.pone.0044181

**Published:** 2012-09-06

**Authors:** Janet M. Turan, Rachel L. Steinfeld, Maricianah Onono, Elizabeth A. Bukusi, Meghan Woods, Starley B. Shade, Sierra Washington, Reson Marima, Jeremy Penner, Marta L. Ackers, Dorothy Mbori-Ngacha, Craig R. Cohen

**Affiliations:** 1 Department of Health Care Organization and Policy, University of Alabama at Birmingham, Birmingham, Alabama, United States of America; 2 Department of Obstetrics, Gynecology, and Reproductive Sciences, University of California San Francisco, San Francisco, California, United States of America; 3 Centre for Microbiology Research, Kenya Medical Research Institute, Nairobi, Kenya; 4 Department of Medicine, University of California San Francisco, San Francisco, California, United States of America; 5 Department of OBGYN and Women’s Health, Albert Einstein School of Medicine, Bronx, New York, United States of America; 6 Aga Khan University Hospital, Nairobi, Kenya; 7 Center for Global Health, Division of Global HIV/AIDS, U.S. Centers for Disease Control and Prevention, Atlanta, Georgia, United States of America; 8 Department of Paediatrics and Child Health, University of Nairobi, Nairobi, Kenya; Indiana University and Moi University, United States of America

## Abstract

**Background:**

Despite strong evidence for the effectiveness of anti-retroviral therapy for improving the health of women living with HIV and for the prevention of mother-to-child transmission (PMTCT), HIV persists as a major maternal and child health problem in sub-Saharan Africa. In most settings antenatal care (ANC) services and HIV treatment services are offered in separate clinics. Integrating these services may result in better uptake of services, reduction of the time to treatment initiation, better adherence, and reduction of stigma.

**Methodology/Principal Findings:**

A prospective cluster randomized controlled trial design was used to evaluate the effects of integrating HIV treatment into ANC clinics at government health facilities in rural Kenya. Twelve facilities were randomized to provide either fully integrated services (ANC, PMTCT, and HIV treatment services all delivered in the ANC clinic) or non-integrated services (ANC clinics provided ANC and basic PMTCT services and referred clients to a separate HIV clinic for HIV treatment). During June 2009– March 2011, 1,172 HIV-positive pregnant women were enrolled in the study. The main study outcomes are rates of maternal enrollment in HIV care and treatment, infant HIV testing uptake, and HIV-free infant survival. Baseline results revealed that the intervention and control cohorts were similar with respect to socio-demographics, male partner HIV testing, sero-discordance of the couple, obstetric history, baseline CD4 count, and WHO Stage. Challenges faced while conducting this trial at low-resource rural health facilities included frequent staff turnover, stock-outs of essential supplies, transportation challenges, and changes in national guidelines.

**Conclusions/Significance:**

This is the first randomized trial of ANC and HIV service integration to be conducted in rural Africa. It is expected that the study will provide critical evidence regarding the implementation and effectiveness of this service delivery strategy, with important implications for programs striving to eliminate vertical transmission of HIV and improve maternal health.

**Trial Registration:**

ClinicalTrials.gov NCT00931216 NCT00931216.

## Introduction

Despite the strong evidence for the effectiveness of anti-retroviral (ARV) drugs for improving the health of HIV-infected women and for the prevention of mother-to-child transmission (PMTCT) [1], [2], HIV-related morbidity and mortality among childbearing women and vertical transmission of HIV from mother to child continue to be major health problems in sub-Saharan Africa [3], [4]. In order to improve health outcomes, women need to successfully navigate a cascade of services including antenatal care (ANC) clinic attendance, acceptance of HIV testing, receipt of results, enrollment in HIV care, acceptance of ARV prophylaxis or treatment, adherence with maternal antiretroviral (ARV) prophylaxis or treatment, adherence to infant ARV prophylactic doses, and early infant HIV testing [5]. Unfortunately, a significant proportion of women and their infants drop out at each step along this cascade thus decreasing the effectiveness of PMTCT programs [6]–[9]. The 2009 Expert Consultation on Implementation Science Research sponsored by the NIH Office of AIDS Research identified as a top priority research on how to promote women’s linkage to and retention in care at each step of this PMTCT cascade [10].

One promising approach for improving linkages to and retention in services for women and infants is to fully integrate HIV care into ANC clinics [11]. Although definitions of “integration” vary, experiences in sub-Saharan Africa suggest that integrating ANC and HIV services may result in a variety of benefits for HIV-positive women and their families; including better uptake of services, more women receiving counselling, reduction of the time to treatment initiation, improved quality of care, and reduction of stigma [12]–[17]. Lack of integration of PMTCT into routine maternal and child health services has been identified as one of the major contributors to drop-off of women and infants at various steps in the PMTCT cascade [18]. On the other hand, the potential downsides of integration in low-resource settings include: increased provider workload in an already overburdened system, increased training needs, lack of space and equipment, lack of staff motivation to provide more services, and even “organizational culture clash” [19]–[21]. From the perspective of clients, it is possible that integration of HIV services into ANC clinics could have negative effects–such as increased wait times–for the majority of clients who are HIV-negative [22].

Since 92% of women in Kenya have at least one antenatal care visit during pregnancy, ANC clinics have become prime locations for expansion of HIV testing and PMTCT services in this country [23]. Due to these efforts, rates of antenatal HIV testing have been increasing over time in Kenya – approximately 73% of pregnant women were tested during 2008–2009 [23]; however, only an estimated 72% of pregnant women who tested HIV-positive received antiretroviral medications for PMTCT in 2009 [24]. Antenatal care has been modified to include PMTCT services, but generally has not included comprehensive HIV care and treatment for pregnant women. Comprehensive HIV care and treatment encompasses the clinical and social components necessary for the highest quality of care, including opportunistic infection prophylaxis, tuberculosis (TB) diagnosis and treatment, WHO clinical staging for HIV, highly active antiretroviral therapy (HAART), CD4 count monitoring, relevant laboratory tests (such as complete blood counts, creatinine, liver enzymes, etc.), adherence counseling, peer education, and access to support groups. In most ANC clinics in Kenya, HIV-positive pregnant women are normally referred to a separate HIV clinic for care and treatment (either located elsewhere on the grounds of the health facility or at another health facility), which may operate at different times and days than the ANC clinic.

As elsewhere, the Kenyan national guidelines for PMTCT–including recommendations for infant feeding, CD4 count monitoring, and HAART initiation–have been evolving over time. In August 2010 the Kenyan PMTCT guidelines were updated [25]. These guidelines recommended earlier initiation of HAART for a larger group of HIV-positive pregnant women (WHO clinical stage III or IV regardless of CD4 count OR WHO clinical stage I or II with CD4 count <350/mm^3^, compared with previous recommendations of WHO clinical stage of III or IV OR CD4 count <200/mm^3^) to benefit both the health of the mother and prevent HIV transmission to her child during pregnancy and breastfeeding. The ARV prophylaxis regimen guidelines were also changed in 2010, to start at 14 weeks or at first contact thereafter, compared with previous guidelines that recommended initiating prophylaxis at 28 weeks. The new PMTCT guidelines also include provision of ARV prophylaxis (zidovudine and lamivudine) to the mother for 7 days after the delivery and daily infant prophylaxis with nevirapine (NVP) monotherapy until one week after breastfeeding cessation if mother is not on HAART or up to 6 weeks of age if mother is on HAART.

## Methods

The protocol for this trial and supporting CONSORT checklist are available as supporting information; see Checklist S1 and Protocol S1.

### Ethics Statement

This study was approved by the Committee on Human Research of the University of California, San Francisco (FWA00000068) and the Ethical Review Committee of the Kenya Medical Research Institute (FWA00002066). The study protocol was also approved by the Associate Director for Science at the US Centers for Disease Control and Prevention. All women participating in the study gave written informed consent for the use of their de-identified data in the evaluation. Participation in the study did not require additional research activities beyond women’s regular antenatal and HIV care. This clinical trial is registered at clinicaltrials.gov NCT00931216.

### Objectives

The primary aim of the Study of HIV and Antenatal care Integration in Pregnancy (SHAIP) is to test if an integrated approach to ANC, PMTCT, and HIV care and treatment provision is an effective service model for low-resource health facilities in rural Nyanza Province, Kenya, with implications for other sites in Kenya and sub-Saharan Africa. In this study, “service integration” has been defined as integrating HIV care and treatment services, including initiation and provision of HAART for eligible women, into existing ANC and PMTCT services, with all services provided by the same health care provider in the same room during ANC visits for the duration of the pregnancy and until a definitive HIV diagnosis of the child (up to 18 months), after which point the woman and infant, if HIV-infected, would be referred to the HIV clinic. Women and infants who presented for HIV care were enrolled on the same day or at the subsequent visit.

We hypothesized that the integrated model would lead to increased uptake of HIV care and treatment for women and infants and thus improved maternal health outcomes and reduced vertical transmission of HIV, as compared to a non-integrated model. While experience and related studies have indicated that integration of services may improve uptake and utilization of HIV care and treatment by pregnant women, full integration needs rigorous evaluation before adoption on a wider scale [12], [20], [26]–[29]. Thus, in order to ensure provision of the highest quality ANC, PMTCT, and comprehensive HIV care and treatment to pregnant women, a new service model should have strong evidence supporting its effectiveness.

### The Setting

In March 2005, the Kenya Medical Research Institute (KEMRI) and University of California, San Francisco (UCSF), supported by the President’s Emergency Plan for AIDS Relief (PEPFAR)/Centers for Disease Control (CDC), launched Family AIDS Care and Education Services (FACES) in Nairobi and Kisumu, Kenya [30]. As one of the PEPFAR local partners in Kenya, FACES works to support the Kenyan Ministries of Health (MoH) in the implementation of quality HIV services including PMTCT at existing public and private health facilities; by providing training, clinical mentorship, logistical support, community engagement activities; as well as employing lay health workers based at peripheral sites and provision of salary support for MoH health workers. FACES currently supports 123 PMTCT sites in Nyanza Province.

As of 2008, HIV prevalence in Nyanza Province was estimated at 16.0% of reproductive-aged women, which is the highest HIV prevalence in the country and more than double the national average [23]. The study sites are located in the southern part of Nyanza Province, bordering Tanzania to the south and covering a third of Kenya’s shoreline of Lake Victoria. The main economic activities in this area are fishing and farming.

### Study Design

SHAIP is a prospective cluster randomized trial. This design was chosen because service integration is an intervention that is carried out on a clinic-level, rather than at an individual patient level; it would be practically impossible to randomize individual women at a given clinic to receive either fully integrated (FI) or non-integrated (NI) services. Outcomes for women and infants attending FI clinics will be compared to those of women and infants at NI clinics covering the time period from June 2009 - March 2012. Although clients and providers could not be blinded as to the type of services delivered, study investigators are blinded in terms of knowledge of any outcomes by study arm until locking of the study database.

### Randomization

**Figure 1:**
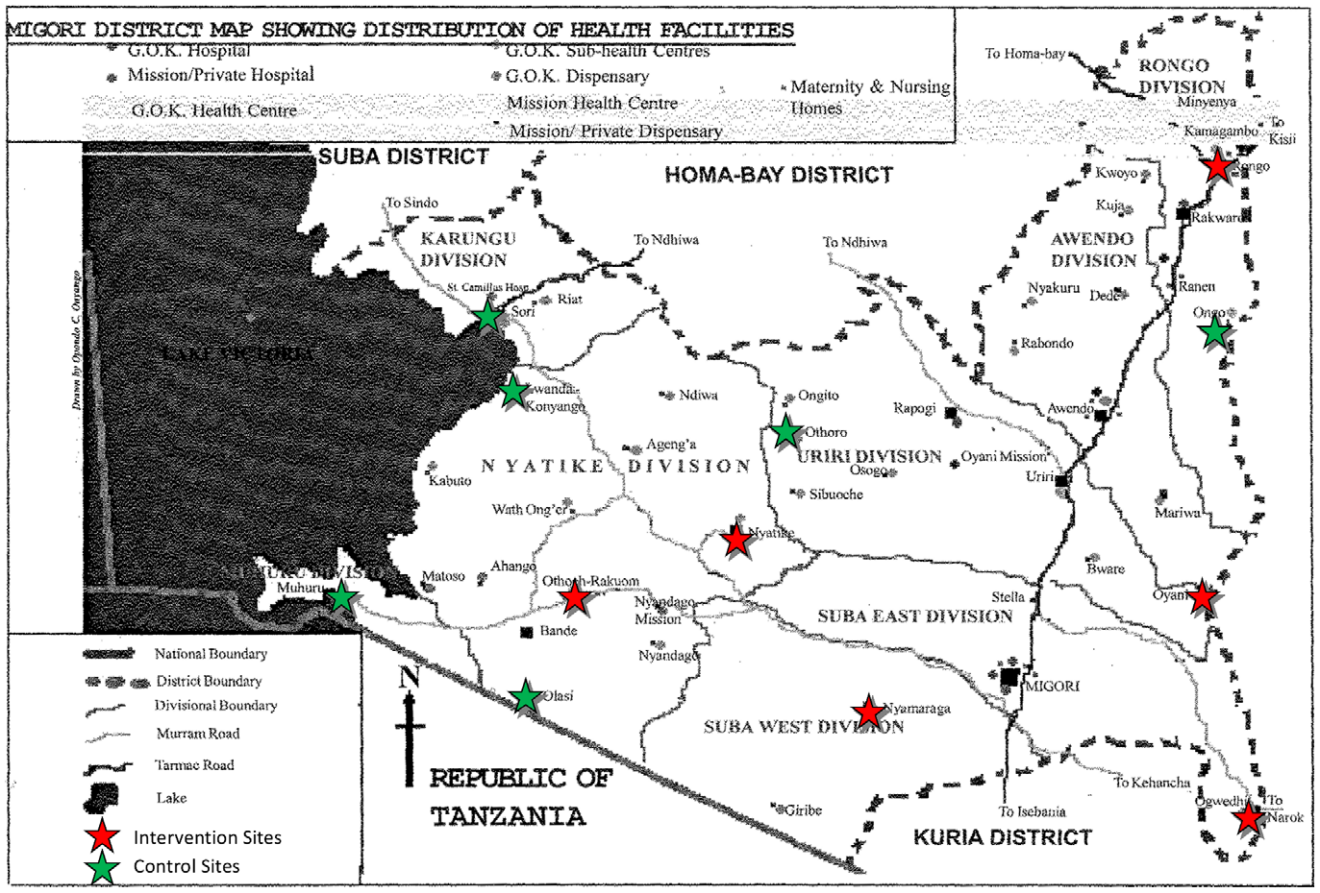
Map of SHAIP Study Sites. This map of the Greater Migori District shows the location of control (NI, indicated by green stars) and intervention (FI, indicated by red stars) health facilities.

Prior to study initiation, potential sites (government health facilities in the study area) were assessed to determine the size of the facility, staffing, patient load, and available services. Inclusion criteria for sites included the following: 1) providing ANC services, 2) providing HIV testing and ARV services for pregnant women, and 3) an average of at least 20 new ANC clients per month. Study sites included all the government health facilities that met these criteria and were also either providing or scheduled to begin providing HAART services in these districts by June 2007 (excluding Migori District Hospital, which was much larger in terms of staffing and patient volume, and thus very different from other health facilities in the districts, and would have created imbalance in the study arms) (See Table 1). Twelve facilities were categorized by facility type, as either “Health Center or Dispensary” (N = 8) or “Hospital” (N = 4). Within these strata, each clinic represented a cluster, and was randomized to either control (NI) or intervention (FI) using the ACluster software [31]. Prior to beginning study enrollment, each site had to have begun providing comprehensive HIV care and treatment services including HAART; site staff had to have completed the study training program, including research ethics; and a site initiation visit had to have been completed by the external monitor. A map of Greater Migori District, showing the locations of control (NI) and intervention (FI) sites is presented as Figure 1.

**Table 1:** Characteristics of the twelve clinic sites.

Site	Type of Facility	NI vs FI	# cliniciansat ANC	# non-cliniciansat ANC	# ANC clientsin June 2010	Location of HIV clinic vis-à-vis ANC clinic	Date of Study Initiation	HAART first became available	Baseline indicators from FACES program data (Dec 08– May 09)		
									% who received PMTCT prophy-laxis	% who received HAART^b^	% of infants who received prophy-laxis
1	Hospital	FI	4	2	248	Different building	Jun 2009	May 2005	76.4%	23.6%	100.0%
2	Hospital	FI	1	3	102	Same building	Jun 2009	Jun 2005	92.9%	7.1%	71.4%
3	Health Center	FI	2	1	122	Same room^a^	Jul 2009	Aug 2008	95.2%	4.8%	100.0%
4	Health Center	FI	2	2	62	Same building	Nov 2009	Jun 2009	96.3%	7.4%	92.6%
5	Dispensary	FI	2	2	137	Same building	Aug 2009	Nov 2008	87.5%	9.4%	96.9%
6	Dispensary	FI	1	1	37	Same room^a^	Sep 2009	Feb 2008	84.0%	4.0%	88.0%
7	Hospital	NI	1	2	69	Different building	Jun 2009	Aug 2006	77.3%	22.7%	100.0%
8	Hospital	NI	1	1	94	Same building	Feb 2010	Aug 2007	100.0%	0.0%	100.0%
9	Health Center	NI	1	1	91	Same building	Jun 2009	Apr 2007	78.1%	21.9%	81.3%
10	Health Center	NI	2	1	139	Same building	Jun 2009	Jun 2008	91.7%	8.3%	95.8%
11	Dispensary	NI	1	1	85	Same building	Aug 2009	May 2008	88.9%	5.6%	74.1%
12	Dispensary	NI	1	3	75	Same building	Nov 2009	Nov 2008	95.8%	0.0%	100.0%

Full Integration (FI), Non Integration (NI), Antenatal care (ANC).

a In some facilities the HIV and the ANC clinics are conducted in the same room, however, these separate clinics are run by different health care providers.

b % of women on HAART presented here is among all HIV+ women, not just those who are eligible.

Table 1 also shows baseline measures of the proportions of a) HIV+ women receiving PMTCT prophylaxis other than HAART, b) HIV+ women receiving HAART, and c) HIV-exposed infants receiving prophylaxis, using data from program PMTCT reporting tools for the study sites during the six months prior to study initiation in June 2009 (December 2008–May 2009). These proportions were very similar for FI and NI sites at baseline before study enrollment began: 87.3% FI vs. 86.0% NI for PMTCT prophylaxis received; 11.6% FI vs. 12.3% NI for HAART received; and 95.1% FI vs. 91.1% NI for infant prophylaxis received. It should be noted that these proportions are based on monthly data from clinic registers and may not reflect actual use/adherence.

### Participants

**Figure 2:**
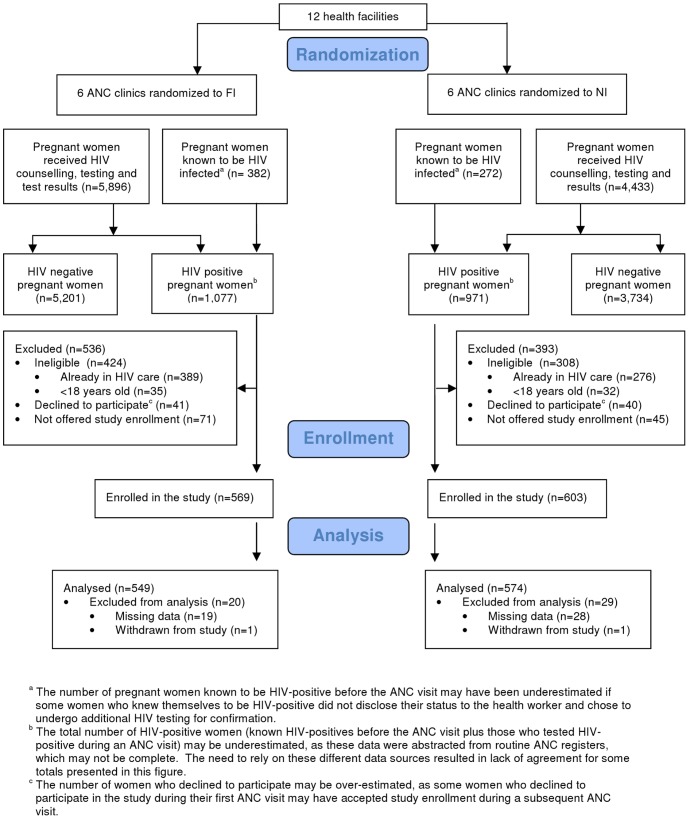
Consort Diagram. The flow diagram of clusters and individuals through the cluster randomized trial. A total of 1,172 HIV-positive pregnant women were enrolled in the study during the period June 2009–March 2011.

The study population included all pregnant HIV-positive women, 18 years and older who were not currently enrolled in HIV care and treatment, as well as infants born to the women enrolled in the study. Eligible women were introduced to the study at the ANC clinic and if they were interested in participation they were taken through an informed consent process in a private location by a trained lay health worker. A total of 1,172 HIV-positive pregnant women were enrolled in the study during the period June 2009–March 2011 (See Figure 2). Of a total of 1,200 eligible women who were offered study enrollment, 81 (6.8%) declined to participate in the study.

### Outcome Measures

The main study outcome measures are rates of maternal enrollment in HIV care and treatment during the 12-month follow-up period, infant HIV testing uptake by 3 months of age, and HIV-free infant survival at 6 months of age. Maternal enrollment in care is assessed by the existence of a completed HIV care enrollment form for the woman in the FACES electronic medical record system, and by calculating time from the date of study enrollment to the date of enrollment in HIV care. Infant testing uptake by 3 months after the birth is measured using a combination of medical record data and study tracking tools. Mothers were encouraged to bring their infants to the clinic for HIV testing at 6 weeks of age and 6 weeks after the complete cessation of breastfeeding, and the FACES program attempted home visits for HIV-exposed infants who were not brought to the facility for HIV testing by 3–6 months after the birth (see below). HIV-free survival at around 6 months of age is calculated by assessing the numbers of infants who were not lost-to-follow-up and were still alive and had not tested HIV-positive at around 6 months of age, based on data from medical records and home visits after the birth.

Infant HIV-Polymerase Chain Reaction (PCR) testing was done on dried blood spots using Roche DNA PCR version 1.5 (Roche Diagnostic System). Infant samples were collected from skin prick and coated onto filter paper. The spotted filter papers were allowed to dry for at least 4 hours at room temperature and placed in individual zip locked bags containing a silica desiccant. All these samples were then transported to the KEMRI-CDC HIV-R laboratory in Kisumu for PCR testing. This laboratory handles all Infant HIV-PCR tests for Nyanza Province. Specimens for CD4 testing were collected in EDTA whole blood collection tubes. CD4/CD3 fluorescent labeled monoclonal antibodies (Becton Dickinson Biosciences) were used to quantify the CD4 absolute count using BD FACSCount™. Additional outcomes to be assessed for HIV-positive women include: receipt of PMTCT prophylaxis, initiation of HAART (among HAART-eligible women), time to initiation of HAART, changes in CD4 counts, and retention in and adherence to HIV care. Samples for CD4 counts are ideally collected at the time of study enrollment and every six months or more frequently, if clinically indicated. Changes in women’s CD4 cell counts from baseline (study enrollment) to approximately 6 months following study enrollment will be assessed using medical record data. Monthly tracking tools and electronic patient records are used to collect data on enrollment in HIV care, initiation of HAART, retention in care, and number of missed visits. Additional study outcomes to be examined for infants include enrollment in HIV care and uptake of HAART, if HIV-positive.

### Sample Size and Power Calculations

From preliminary site assessments of the clinics conducted in 2006, it was estimated that 6,564 new ANC clients would be seen in a 12-month period at the 12 study sites. It was expected that an average of 80% of pregnant women would agree to HIV testing. At the time when the sample size calculations were performed, the most recent estimate of HIV prevalence among pregnant women in Nyanza Province was 25% [32]. These assumptions resulted in estimates of approximately 1,313 women testing HIV-positive with a previously unknown HIV-serostatus, and 3,938 being HIV-negative after the first ANC visit. Assuming that 90% of the women who tested HIV-positive agreed to participate in the study, we estimated being able to enroll 1,182 HIV-positive pregnant women in the study over a period of 12 months.

Power calculations were made based on estimated differences in vertical transmission rates in the two arms of the study, accounting for stratification, based on prior published transmission rates using similar antiretroviral protocols in Thailand and Cote-D’Ivoire for reference [33]–[35]. With a sample size of 12 clusters, 591 HIV-positive women per study arm, and an average of 98 HIV-positive women per health facility (average in the Hospital stratum of 146 women per facility and average in the HC/Dispensary stratum of 75 women per facility), we calculated that we would have 80% power to be able to detect a minimum odds ratio of 2.02 for vertical transmission, if we assumed a conservative intracluster correlation coefficient (ICC) of .01. We also calculated our minimum detectable effect size with a range of ICCs [36] and found that the minimum detectable odds ratio ranged from 1.81 for a very low ICC of .005, to 3.75 if the ICC is as high as .05.

With 99% power, this sample size allows us to detect a mean difference of 50 cells in CD4 counts between groups, using an estimated standard deviation of 142 cells and an intra-cluster correlation coefficient of .01.[37]–[39] Our power will be 90% if the ICC is .025, and 80% if the ICC is as high as .037. This sample size also provides us with exceptional power to examine the other study outcomes including women’s enrollment in HIV care, pediatric HIV testing uptake, and loss-to-follow for those who enroll in HIV care.

### Description of Procedures

Health care providers at both FI and NI facilities were trained and supported to provide high quality ANC, PMTCT, and HIV care and treatment services throughout the duration of the study. Refresher trainings were provided on a regular basis, and were quickly organized in response to significant staff turnover at a site and/or changes to the Kenyan national PMTCT guidelines. Study-specific training sessions for the FI and NI clinics included study procedures, correct filling of patient medical record forms, clinic flow and logistics for FI or NI services, informed consent procedures, and participant eligibility criteria. Clinical trainings covered the components of high quality ANC, PMTCT, and HIV treatment for pregnant women; early infant diagnosis; and care and treatment for HIV-exposed infants and HIV-infected children.

**Figure 3:**
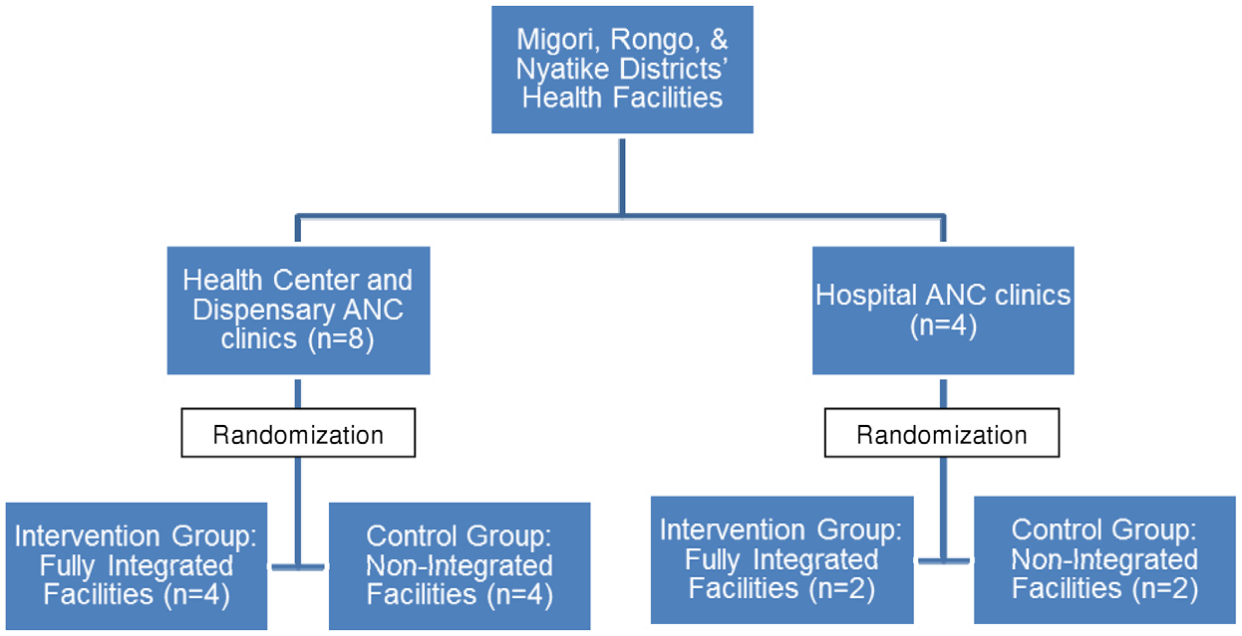
Randomization of clinic sites. Twelve facilities were categorized by facility type, as either “Health Center or Dispensary” (N = 8) or “Hospital” (N = 4). Within these strata, each clinic represented a cluster, and was randomized to either control (Non-Integrated) or intervention (Fully Integrated) using the ACluster software.

**Figure 4:**
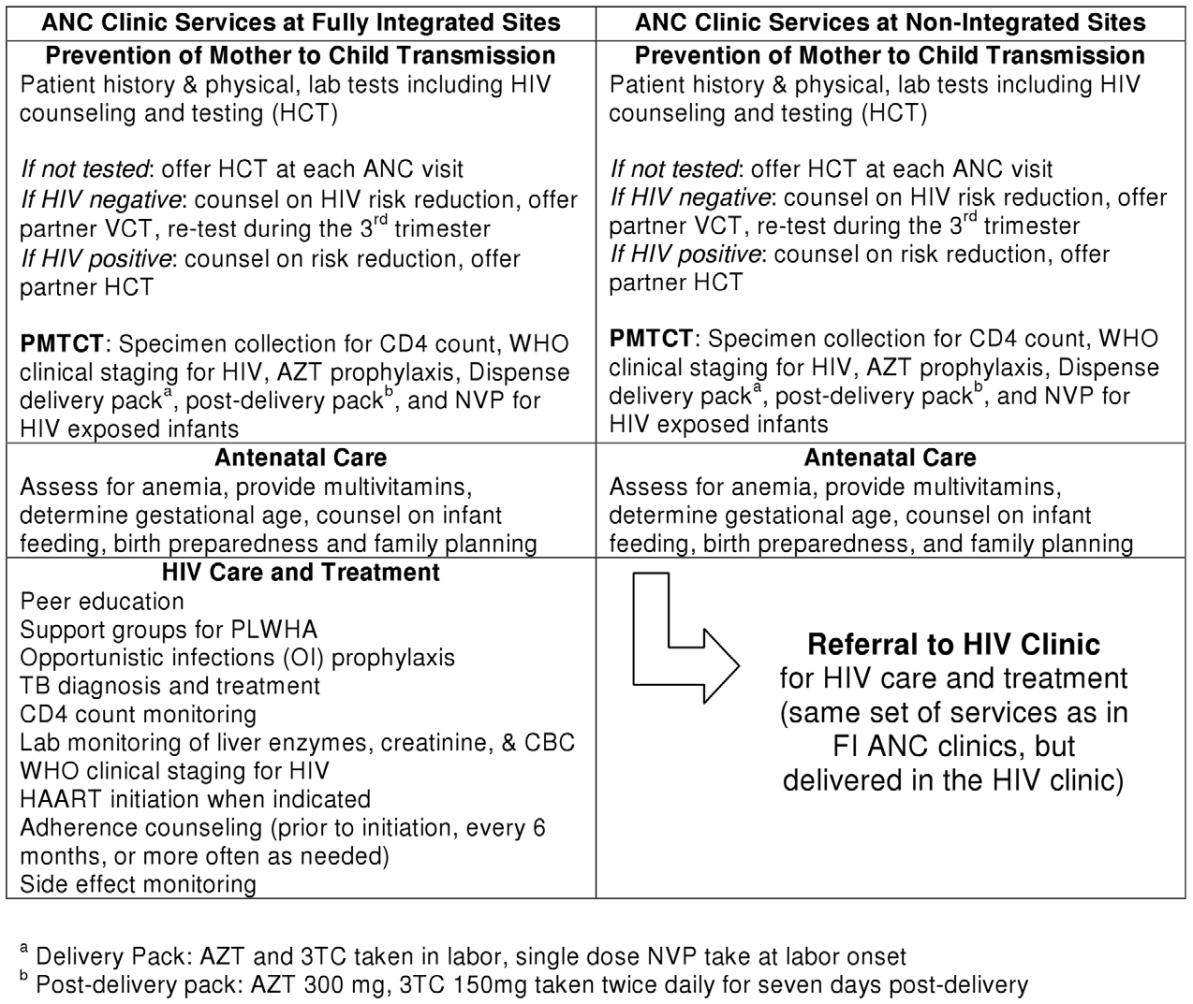
Clinical services provided in the ANC Clinic at Fully Integrated sites and Non-Integrated sites. ANC, PMTCT, and HIV services available at the Fully Integrated sites and Non-Integrated sites based on Kenyan National guidelines.

The package of ANC, PMTCT, and HIV care and treatment services offered at the sites has followed current Kenyan national guidelines in both study arms for the duration of the study, with the only difference in care being the clinic location of HIV care and treatment services for pregnant and postpartum women (ANC clinic versus the HIV clinic). At the NI clinics, HIV-positive women were given ARVs for PMTCT, clinical staging to determine eligibility was conducted, and a specimen was taken for a CD4 count. They were also given a referral form to enroll in the HIV care and treatment clinic. At the FI clinics, HIV positive women were provided all ANC, PMTCT, and HIV services in the ANC clinic including HAART for women who were eligible. (See Figures 3 and 4).

As mentioned previously, over the course of the study, national guidelines concerning infant feeding, CD4 count monitoring, and HAART initiation were updated. The sites complied with each set of changes shortly after the national guidelines on PMTCT were announced with the exception of daily NVP for the infant, which was rolled out to the study sites in October 2010. The changes were implemented at all study sites simultaneously.

### Data Collection Methods

Quantitative data on women and infants were collected prospectively as part of their routine clinic care. FACES uses an electronic medical record system using the Open Medical Record System (OpenMRS®) platform. In the study area, paper medical record forms were filled out by clinical staff at the health facilities. Data clerks entered these forms into laptop computers on a regular basis, and subsequently these data were merged with the larger FACES database. This dataset includes detailed information on socio-demographics, health indicators (CD4 counts, WHO Staging, vertical transmission, etc.) and use of specific HIV and maternity services by HIV-positive pregnant women who attend FACES-supported sites. For each study participant, data were abstracted from the FACES database until her infant was approximately six months old (12 months after study enrollment). In addition, study registers were used to monitor study recruitment, enrollment in HIV care, infant HIV testing uptake and results, and for follow-up of women who do not return to the health facility for a return visit.

Regardless of study facility or arm, mothers were encouraged to bring their infants to the clinic for HIV testing at 6 weeks of age and 6 weeks after the cessation of breastfeeding. In the initial study period, if a woman had not brought in her child for testing by the time the child was 7 months old, a FACES staff member conducted a home visit to ascertain whether the infant had been tested or not and to encourage the family to bring the infant in for testing, and for the mother and other family members to enroll in HIV care and treatment. Starting in November 2010, FACES began to support home-based testing services for HIV-exposed infants at the 12 facilities engaged in the SHAIP trial. FACES staff were trained to offer dried blood spot (DBS) infant testing during a home visit for HIV-exposed infants who had not been brought to the clinic by the age of three months. During these home visits, mothers are asked for permission to test their infant for HIV at the home using a heel-stick DBS or are referred to the clinic.

Site assessments of the clinics were performed every six months to monitor service delivery parameters and various aspects of service integration, including fidelity to the randomized service model. These assessments were also used to identify needs for refresher trainings due to staff turn-over and to make other changes such as addressing insufficient supply of commodities such as HIV test kits and condoms.

### Monitoring

External monitoring for this study was conducted by FHI 360, including initial site assessment visits, interim monitoring visits, and study close-out visits. Internal monitoring such as form completion and quality control of data entry were conducted on a monthly basis by the study team. Data collection was completed as of March 31, 2012 and the database was closed as of June 29, 2012.

### Statistical Methods

Analyses will examine the effects of integration on service uptake and retention at each step of the PMTCT cascade, as well as effects on vertical transmission of HIV and maternal health outcomes. Analyses will mainly be performed at the cluster (health facility) level and any individual-level analyses will take into account cluster effects [40]. Primary analyses will be based on the “intention-to-treat” (ITT) principle, with facilities randomized to the intervention group classified as delivering “fully integrated services” regardless of whether or not individual women actually received integrated services. In addition, we will perform an according-to-protocol (ATP) analysis, taking into consideration the assessments of integration performed every six months at each site. Assessment data will be used to compute an “integration index” which takes into account various aspects of the integration of services including the location and functioning of trained staff, services, infrastructure, equipment, and supplies. This index will be used to assess variability in the service delivery models for both intervention and control clinics.

In this initial paper, we compared participant characteristics between participants enrolled at intervention (FI) and control (NI) sites using proportions for categorical variables and means or medians (as appropriate) for continuous variables. An assessment of the distribution (normally or skewed) of continuous variables was performed. Socio-demographic and clinical characteristics of participants enrolled at intervention and control sites were compared using methods to account for the similarity (intracluster correlation) between participants enrolled at each site. Statistical tests used included chi-square tests for categorical variables and Student’s t-tests for continuous variables, and were adjusted for clustering effects using the clttest and clchi2 routines in Stata version 11 (StataCorp., College Station, TX, USA).

**Table 2:** Baseline characteristics of study participants (N = 1121)^a^.

	FI (n = 549)	NI (n = 572)	All (N = 1121)	All n	Test Statistic^b^	P value
**Mean age in years (SE)**	25.4 (0.2)	25.2 (0.2)	25.33 (0.2)	1121	t = −0.64	0.534
**Education, n (%)**				1102	Chisq = 0.38	0.537
None^c^ or Some Primary	461 (85.5%)	495 (87.9%)	956 (86.8%)			
Some Secondary or more	78 (14.5%)	68 (12.1%)	146 (13.2%)			
**WHO stage, n (%)**				1027	Chisq = 5.04^d^	0.169
WHO Stage 1	317 (63.9%)	430 (81%)	747 (72.7%)			
WHO Stage 2	72 (14.5%)	34 (6.4%)	106 (10.3%)			
WHO Stage 3 or 4^e^	29 (5.9%)	7 (1.3%)	36 (3.5%)			
Not Staged	78 (15.7%)	60 (11.3%)	138 (13.4%)			
**Mean Baseline CD4 (SE)** f	493.2 (20.7)	523.6 (19.4)	508.2 (14.2)	608	t = 1.07	0.309
**Baseline CD4, n (%)**				608	Chisq = 1.92	0.165
CD4≤350	107 (34.9%)	87 (28.9%)	194 (31.9%)			
CD4>350	200 (65.1%)	214 (71.1%)	414 (68.1%)			
**Marital status, n (%)**				1065	Chisq = 0.16	0.923
Married	443 (84.4%)	455 (84.3%)	898 (84.3%)			
Single/Separated/Divorced	46 (8.8%)	43 (8.0%)	89 (8.4%)			
Widowed	36 (6.9%)	42 (7.8%)	78 (7.3%)			
**Partner tested for HIV (woman’s report), n (%)**				821	Chisq. = 0.06	0.970
Yes	78 (18.4%)	75 (18.9%)	153 (18.6%)			
No	190 (44.7%)	172 (43.4%)	362 (44.1%)			
Don’t know	157 (36.9%)	149 (37.6%)	306 (37.3%)			
**Partner’s HIV Status (woman’s report), n (%)**				471	Chisq = 0.66	0.718
HIV Positive	38 (15.6%)	41 (18%)	79 (16.8%)			
HIV Negative	29 (11.9%)	17 (7.5%)	46 (9.8%)			
Don’t know	176 (72.4%)	170 (74.6%)	346 (73.5%)			
**Mean No. Pregnancies (SE)**	3.1 (0.1)	3.1 (0.1)	3.1 (0.1)	1081	t = 0.09	0.930
**Mean Gestational Age at ANC Visit (weeks) (SE)**	25.9 (0.3)	25.3 (0.3)	25.6 (0.2)	1066	t = −1.38	0.197

Full Integration (FI), Non Integration (NI), Antenatal care (ANC).

a Individual variables have n’s less than the overall N due to missing values or non-applicable cases.

b Statistical tests (Chi Square & T-test) were adjusted for clustering using the clttest and clchi2 routines in Stata.

(StataCorp., College Station, TX, USA).

c Less than 1% of the participants had no education.

d If we exclude those women who were not staged, Chisq = 4.90, p = .086.

e Less than 1% of the participants were at WHO stage 4.

f Baseline CD4 counts were extracted from the study register; however, baseline CD4 counts were not obtained from all participants. Baseline CD4 counts were available for 307 FI clients and 301 NI clients. All other data were extracted from the participant’s electronic medical record.

## Results

### Baseline Characteristics of Participants

Table 2 presents the baseline characteristics of pregnant women enrolled in the study by study arm. The distributions of the continuous variables did not deviate significantly from the normal distribution and thus t-test methods could be used to test the differences between the two groups. The intervention and control cohorts were similar at baseline with respect to their mean age, the patient’s report of whether or not her male partner had been tested for HIV, patient-reported sero-discordance of the couple, marital status, education, obstetric history, and baseline CD4 count. Participants in the intervention group were somewhat more likely to have WHO stage 3 or 4 disease and less likely to have WHO stage 1 disease than participants in the control group, although these differences were not statistically significant. The majority of participants were relatively young women (median age 25.3 years), had low educational level (only 13.2% had more than a primary school education), and were married (84.3%).

### Implementation Challenges

Conducting implementation science research in the context of a dynamic HIV service program has presented both methodological and logistical challenges. Study initiation was delayed for two years after initial approval from the funder due to bureaucratic hurdles; therefore some aspects of the service environment had already changed between study design and implementation. In addition, it would have been ideal if all clinic sites would have begun the study simultaneously. However, at the time of study initiation, not all of the sites met all of the protocol requirements for site initiation. Due to a combination of issues listed above, study initiation was staggered across the 12 sites over a nine-month period. However, the timing of study initiation was balanced between intervention and non-intervention clinics. Study clinics, like other clinics in this region, regularly faced challenges to providing high quality care. During the course of the study there were periods of staff turnover, staff shortages, lack of HIV test kits or other critical clinic supplies and equipment. For some study participants, baseline CD4 counts and follow-up CD4 counts could not be obtained due to lack of laboratory supplies and inadequate transportation systems to get lab specimens to the district laboratories. Several sites experienced complete staff turnover up to three times during a two-year period and nearly all sites experienced more than 40% staff turnover during the study period. Special challenges arose in a few cases when a staff member from a control site was re-assigned to an intervention clinic and vice versa. This type of challenge is inherent in “real world” implementation trials where patient care must be balanced with the study protocol [41].

The diversity of the study sites, which are dispersed over a wide geographical area, posed an additional challenge. For example, one site sits on the banks of Lake Victoria and the population engages in fishing-related activities that involve extensive travel, making ongoing follow-up of clients difficult. Other sites serve predominately farming communities, which tend to be more stable. Many sites serve mostly patients from the Luo ethnic group, while others serve patients from a variety of ethnic groups. However, the variations in study site and populations increase external validity and wide applicability.

Recruitment of HIV-positive pregnant women who were not enrolled in HIV care into the study was slower than expected. This was partially due to the fact that decentralization and scale up of HIV care and treatment services in the districts during the study period led to lower than anticipated numbers of eligible pregnant women (not already enrolled in HIV care and treatment) at the study sites. Additionally, HIV prevalence among pregnant women was lower than expected; 19% of ANC attendees at all FACES-supported health facilities in Nyanza Province tested HIV-positive in 2010, in comparison to the 25% prevalence anticipated at the time the study protocol was written [42]. Thus, study enrollment took 21 months, instead of the expected 12 months.

In order to improve early uptake of HIV exposed infant testing, during the study period the sites began conducting home visits for women who have not returned to the health facility of ANC care with their infant by 3 months of age, instead of 7 months of age as indicated in the original study protocol. At the same time, these visits were modified to include home-based HIV infant testing, whereas in the earlier period women were referred to the nearest health facility for infant testing. We anticipate that both of these changes will increase uptake of early infant diagnosis at both FI and NI sites, and these changes will be factored into the analyses.

## Discussion

The SHAIP Trial will provide important data about the effects of the full integration of ANC, PMTCT, and HIV care and treatment services on maternal and infant health outcomes. Many other models of integration exist–in other contexts the term “integration” has referred to a variety of other service combinations such as: adding PMTCT services to existing ANC services, integrating HIV care into general primary care, and having HIV care providers attend ANC clinics on specific days [18], [26], [43]. We chose to investigate the effects of fully integrated HIV care and treatment into ANC clinics because experience in sub-Saharan Africa suggests that this model might result in the most improved uptake of HIV care and treatment services by pregnant women and better outcomes for women and infants [14], [15], [43]. In Zambia, for example, researchers conducted a stepped wedge evaluation and found that integration of ANC and HIV services doubled the proportion of treatment-eligible women enrolling in HIV care, as well as doubling the proportion of treatment-eligible women initiating HAART while pregnant [12]. In South Africa, researchers conducted a pre-post evaluation and found that strengthening linkages and integrating key components of ARV treatment within antenatal care significantly reduced the time to treatment initiation [43].

This study will contribute to the growing body of research on integration of HIV services into other primary health care services globally [12], [17], [27], [44], [45]. Although recent systematic reviews of sexual and reproductive health and HIV integration intervention studies have found that integration is feasible and that the majority of studies report positive effects on outcomes [28], [29]; they also point out the dearth of rigorous study designs examining this issue [28], [46]. The SHAIP Trial is the first cluster-randomized trial of ANC and HIV care service integration in sub-Saharan Africa.

Sub-studies have been conducted during the course of the SHAIP Trial to examine potential downsides of ANC and HIV service integration from the perspectives of patients and providers. A study of patient satisfaction at 5 fully integrated and 4 non-integrated sites found that integration resulted in significantly higher satisfaction with the ANC visit for HIV-positive women, without adversely affecting satisfaction for HIV-negative women [47]. Interviews with healthcare providers at SHAIP sites indicated that most providers were supportive of integration and expected positive effects for their clients, but were concerned about effects on workload, disclosure, and the quality of care [48].

The implementation challenges presented above introduce some limitations to the study. Insufficient staffing and supplies at the study facilities at some time periods during the course of the study may mean that the full benefits of services could not always be realized for all women enrolled in the study, thus potentially diluting the effects of service integration. Lack of adequate systems for obtaining baseline and follow-up CD4 counts for pregnant women at the study health facilities may result in inadequate power to examine differences in the maternal health outcome of change in CD4 counts. The relatively small number of sites (12) and their diversity mean that there may be imbalances in the two study arms that may not be captured by available variables. The longer than expected enrollment period for the study means that the analyses will need to take into account changes in national guidelines and program practices during the study period, which will make the analyses and their interpretation more complex.

Although demonstration of cost-effectiveness was not included in the design of this trial, there are indications that the integration model tested may be cost-effective. This model of service integration did not involve adding any additional staff or resources beyond the normal MoH and FACES inputs to these health facilities. Effective operation of both service delivery models (integrated or non-integrated) requires healthcare provider training and ongoing mentorship. The Kenyan MoH has already stated a commitment to integration of HIV and Reproductive Health services and is ready to scale up integration if beneficial effects on health service utilization and outcomes are demonstrated [49].

In addition to the primary results on the effects on health service uptake and health outcomes, this study will include detailed analysis to help understand factors related to successful integration and improvement of ANC, PMTCT and HIV care; such as patient load, facility size, and staff composition. Furthermore, patient characteristics such as age, marital status, and HIV disclosure will be analyzed to elucidate which patients are most influenced by integration. A concurrent study on HIV/AIDS stigma conducted at some of the SHAIP sites [50] will also allow us to examine potential effects of service integration on pregnant women’s fears and experiences of HIV/AIDS stigma in this setting. Overall, the SHAIP study will provide much-needed, high-quality evidence about the popular concept of integration.
